# Observability Transitions in Networks with Betweenness Preference

**DOI:** 10.1371/journal.pone.0156764

**Published:** 2016-06-14

**Authors:** Yang Shunkun, Yang Qian, Xu Xiaoyun, Lu Dan, Li Daqing

**Affiliations:** 1 School of Reliability and Systems Engineering, Beihang University, Beijing, China; 2 Science and Technology on Reliability and Environmental Engineering Laboratory, Beijing, China; Glasgow University, UNITED KINGDOM

## Abstract

A network is considered observable if its current state can be determined in finite time from knowledge of the observed states. The observability transitions in networks based on random or degree-correlated sensor placement have recently been studied. However, these placement strategies are predominantly based on local information regarding the network. In this paper, to understand the phase transition process of network observability, we analyze the network observability transition for a betweenness-based sensor placement strategy, in which sensors are placed on nodes according to their betweenness. Using numerical simulations, we compute the size of the network’s largest observable component (LOC) and compare the observability transitions for different sensor placements. We find that betweenness-based sensor placement can generate a larger LOC in the observability transition than the random or degree-based placement strategy in both model and real networks. This finding may help to understand the relationship between network observability and the topological properties of the network.

## Introduction

Modern society is supported by critical infrastructures such as the Internet, traffic networks, and power grid networks. These critical infrastructures are threatened by cascading failures [1, 2], which can cause incalculable damage. In the attempt to avoid such failures, system state estimation can assist in predicting the dynamical behaviors of these systems [3]. The accurate estimation of system states is important for the early detection of cascading failures. The purpose of network observability analysis is to determine whether the states of a network can be estimated from the currently available measurements, depending on both the number and placement of the sensors used for detection [4]. Therefore, network observability plays a significant role in quantifying and controlling a system. For example, in a power grid network, we can measure the voltage and line current of a node in real time by placing a phasor measurement unit (PMU) [5] on the node, which can help to predict the future state of the system and the possible risks facing it. However, it is not practical to allocate a sensor to every node. Therefore, various optimization techniques have been developed, such as the bisecting search method [6], binary integer programming [7], genetic algorithms [8, 9], particle swarm optimization [10, 11] and tabu search [12].

With the proper selection of the sensor positions, a connected cluster of observable nodes can be formed, which will then increase in size with the further addition of sensors. This process is regarded as a new type of phase transition, termed an observability transition [3]. The network’s largest observable component (LOC) is defined as a measure of the network observability. The LOC increases as more sensors are added to the network. When there are sufficient sensors, the LOC can expand to the full scale of the network. Evaluations of this transition are based on numerical simulations or theoretical analysis. Yang Yang *et al.* found that the random placement of PMUs gives rise to a new type of percolation transition, namely, a network observability transition, and derive the exact analytical solution that describes the size of a network’s LOC for this sensor placement strategy [3]. The observability transition in degree-correlated networks has also been studied. The LOC is larger in negative degree-correlated networks than in those with null or positive degree correlation [13]. Although the network observability transitions associated with random and degree-based sensor placement have been studied, the placement of sensors based on traffic flow has not been addressed. The betweenness of a node measures its importance from the global perspective of traffic on the network; it is proportional to the number of shortest paths passing through the node. Unlike the node degree, the betweenness typically reflects a node’s ability to bridge the entire network.

Motivated by the previous studies discussed above, in this paper, we develop a method of placing sensors based on the betweenness of nodes in the network and study the resulting transition in network observability. We compare the network observability transitions for different placement strategies, and test these results on both model and real networks.

## Methods

In this section, we describe the betweenness-based sensor placement algorithm that we propose to study the network observability. Moreover, we consider two other algorithms as references to illustrate the effect on the network observability of sensor placement based on betweenness. For a given network, we select *N* * Φ directly observable nodes owning sensors initially, and all of their nearest neighbors become also observable; such a node is considered indirectly observable. We denote this fraction of directly observable nodes by Φ(0 ≤ Φ ≤ 1). As only node with a detector and its nearest-neighbors are observable, each node in the network is either directly observable, indirectly observable, or unobservable. In this study, we use numerical simulations to compute the size of the network’s LOC, which is defined as the largest connected component composed of directly or indirectly observable nodes, for various sensor placements.

**Fig 1:**
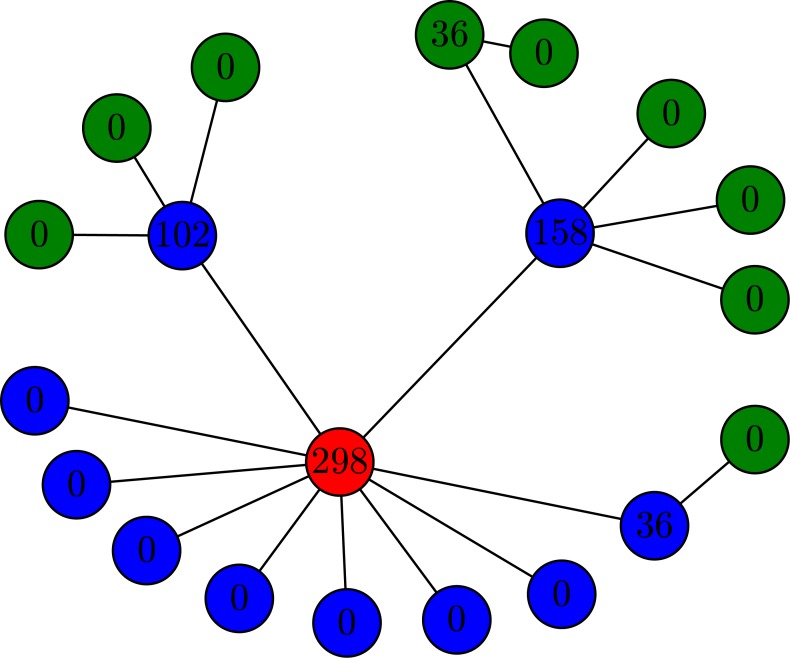
Illustration of betweenness-based sensor placement. The number indicated in each circle is the betweenness of the node. The red circle represents a directly observable node, the blue circles are indirectly observable nodes, and the green nodes are unobservable nodes. Sensors are placed on nodes according to Eq 1. As defined in Ref. [3], in the LOC, nodes with non-zero injection are also observable.

Here, we introduce our method of betweenness-based sensor placement. We place a sensor on node *i* in the network with probability

$$\begin{array}{c} \varphi_{i}=B_{i}^{\alpha }/\sum_{j=1}^{N}B_{j}^{\alpha }, \end{array}$$ (1)

where *B*_*i*_ represents the betweenness of node *i* and *N* is the size of the network. At large *α* (*α* > 0), nodes with high betweenness will have a high probability of being selected. For example, the red node shown in Fig 1 will be allocated a sensor with probability $\varphi_{i}=B_{i}/\sum_{j=1}^{N}B_{j}=298/630=0.473$. We select a certain fraction of the nodes on which to place sensors in accordance with this probability calculation. We compare this method with two other methods, as described below:

1. Random sensor placement: Consider an arbitrary network in which each node is assigned a sensor with equal probability
  $$\begin{array}{c} \varphi_{i}=1/N, \end{array}$$ (2)
  where *N* is the network size. This approach is referred to as random placement.
2. Degree-based sensor placement: We place a sensor on each node in the network with probability
  $$\begin{array}{c} \varphi_{i}=k_{i}^{\alpha }/\sum_{j=1}^{N}k_{j}^{\alpha }, \end{array}$$ (3)
  where *k*_*i*_ is the degree of node *i* and *N* is the network size. This approach is referred to as degree-based sensor placement.

To study the observability transition associated with betweenness-based sensor placement, we calculate the size of the LOC for a given number of sensors. In addition to the size of the LOC, we also consider its diameter to quantify the communication efficiency in the observable component of the network.

## Results

We use numerical simulations to study the size of the LOC for a given fraction of directly observable nodes, Φ(0 ≤ Φ ≤ 1). First, we generate Erdős-Renyí (ER) and scale-free networks [14] to illuminate our methods. In the case of an ER network with *n* nodes, each pair of nodes is connected with probability *p*, and the connection status of each pair is independent of that of all other pairs. The degree distribution of the nodes is as follows:

P(k)=n-1kpk(1-p)n-1-k≈〈k〉ke-〈k〉k!, (4)

where the average degree 〈*k*〉 = (*n* − 1)*p*.

In the case of a scale-free network, we generate the network using the configuration model [15]. To generate the node degrees following a scale-free distribution, a random number *u* is drawn from a uniform distribution between 0 and 1, and this number is then used to generate a new number *k* as the degree of a node using the following formula:

$$\begin{array}{c} k=\frac{m}{u^{1/(\gamma -1)}}, \end{array}$$ (5)

where *m* is the minimum degree and *γ* is the degree exponent. We use Eq 5 to create a degree list, in which each node is repeated a number of times equal to its degree. We then randomly choose pairs from this list and connect the chosen nodes. The chosen pair is removed from the list, and this process is repeated until the list is empty.

**Fig 2:**
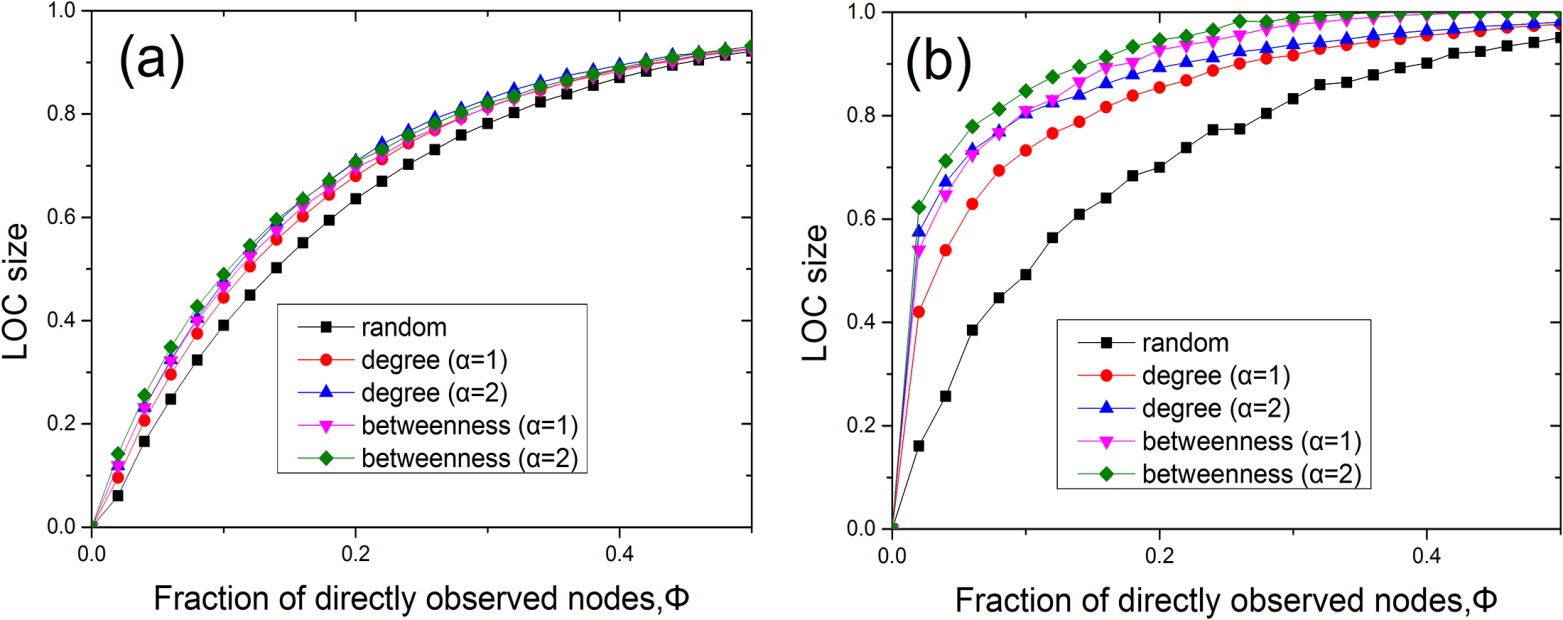
The LOC sizes in ER and scale-free networks of *N* = 10^4^. (a) The LOC size of an ER random graph with 〈*k*〉 ≈ 4 for random, degree-based and betweenness-based sensor placement. (b) The LOC size of a scale-free network generated using the configuration model with *m* = 2, *γ* = 2.5 and 〈*k*〉 ≈ 5.4 for random, degree-based and betweenness-based sensor placement.

**Fig 3:**
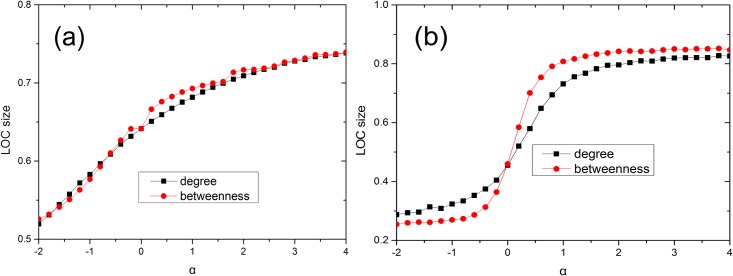
The LOC sizes as a function of *α* in ER and scale-free networks at a given fraction of sensors. (a) The LOC size of an ER random graph with 〈*k*〉 ≈ 4 for degree-based and betweenness-based sensor placement at a given fraction of sensors Φ = 0.2. (b) The LOC size of a scale-free network generated using the configuration model with *m* = 2, *γ* = 2.5 and 〈*k*〉 ≈ 5.4 for degree-based and betweenness-based sensor placement at a given fraction of sensors Φ = 0.1.

**Fig 4:**
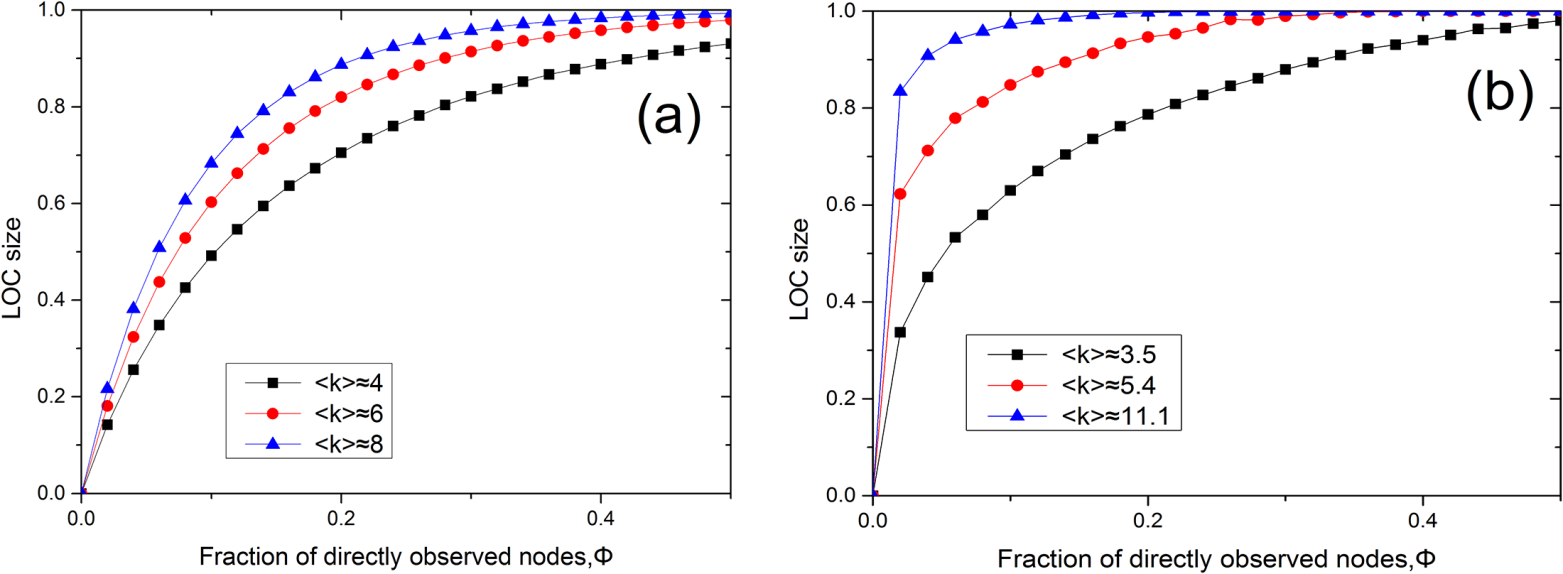
The LOC sizes in ER and scale-free networks under different degree densities. (a) The LOC size of an ER random graph with 〈*k*〉 ≈ 4, 〈*k*〉 ≈ 6, and 〈*k*〉 ≈ 8 for betweenness-based sensor placement (*α* = 2). (b) The LOC size of a scale-free network generated using the configuration model with 〈*k*〉 ≈ 3.5 (*m* = 2, *γ* = 3), 〈*k*〉 ≈ 5.4 (*m* = 2, *γ* = 2.5), and 〈*k*〉 ≈ 11.1 (*m* = 4, *γ* = 2.5) for betweenness-based sensor placement (*α* = 2).

**Fig 5:**
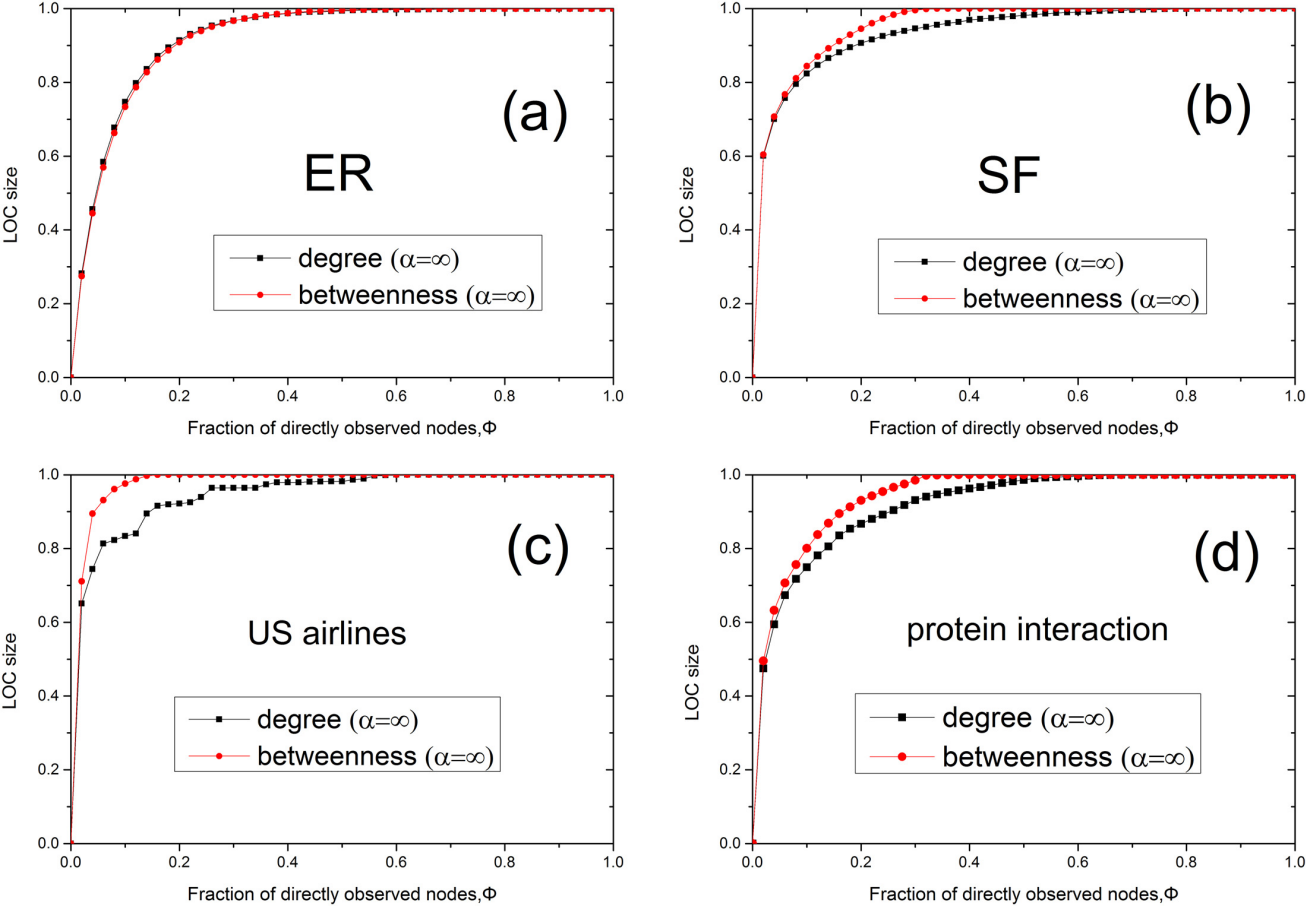
The effect of infinite alpha for degree-based and betweenness-based sensor placement in models and real networks. (a) ER network with 〈*k*〉 = 8. (b) Scale-free network generated using the configuration model with 〈*k*〉 ≈ 5.4 (*m* = 2, *γ* = 2.5). (c) US airlines network. (d) Protein interaction network.

We calculate the LOC size for each model network, namely, ER and scale-free networks, for all three considered methods of sensor placement. In Figs 2 and 3, we present the results of numerical simulations performed on ER (Fig 2(a)) and scale-free (Fig 2(b)) networks for different values of *α* and degree densities, where the LOC size at each Φ value is averaged over 100 realizations. It is evident from Fig 2(a) that the LOC size gradually increases as the fraction of nodes instrumented with sensors is increased. The findings demonstrate that the LOC sizes for degree-based placement and betweenness-based placement are larger than that for random placement when the same number of sensors are placed in the network. These results are consistent with those of a previous study [13]. The degree-based and betweenness-based methods produce similar results in ER networks. Compared with Fig 2(a), in Fig 2(b), we observe that the LOC size increases more abruptly with the addition of only a small fraction of sensors, under betweenness-based placement. Furthermore, the LOC size in the case of betweenness-based placement is significantly larger than that for degree-based placement at the same alpha on SF networks. This is because the high-betweenness nodes play a central role in establishing global connections throughout the network, whereas nodes of high degree do not always serve a similarly role [16]. It is also shown from Fig 3 that betweenness (degree)-based placement with a larger *α* can yield a larger LOC in SF networks for a given Φ. Meanwhile, in Fig 4, we observe that a network with higher average degree can get a larger LOC size. In addition, we investigated the case of *α* = ∞ on models and two real networks (Fig 5). Our results show that for the infinite alpha, the betweenness method and degree-based method generate different LOC clusters in model and real networks. For small occupation fraction of sensors in the model network, due to the randomized structure of configuration model, these two methods lead to similar results. However, essentially, these two methods generate different results due to the distinct role of nodes with large degree or betweenness in most cases including real networks.

**Fig 6:**
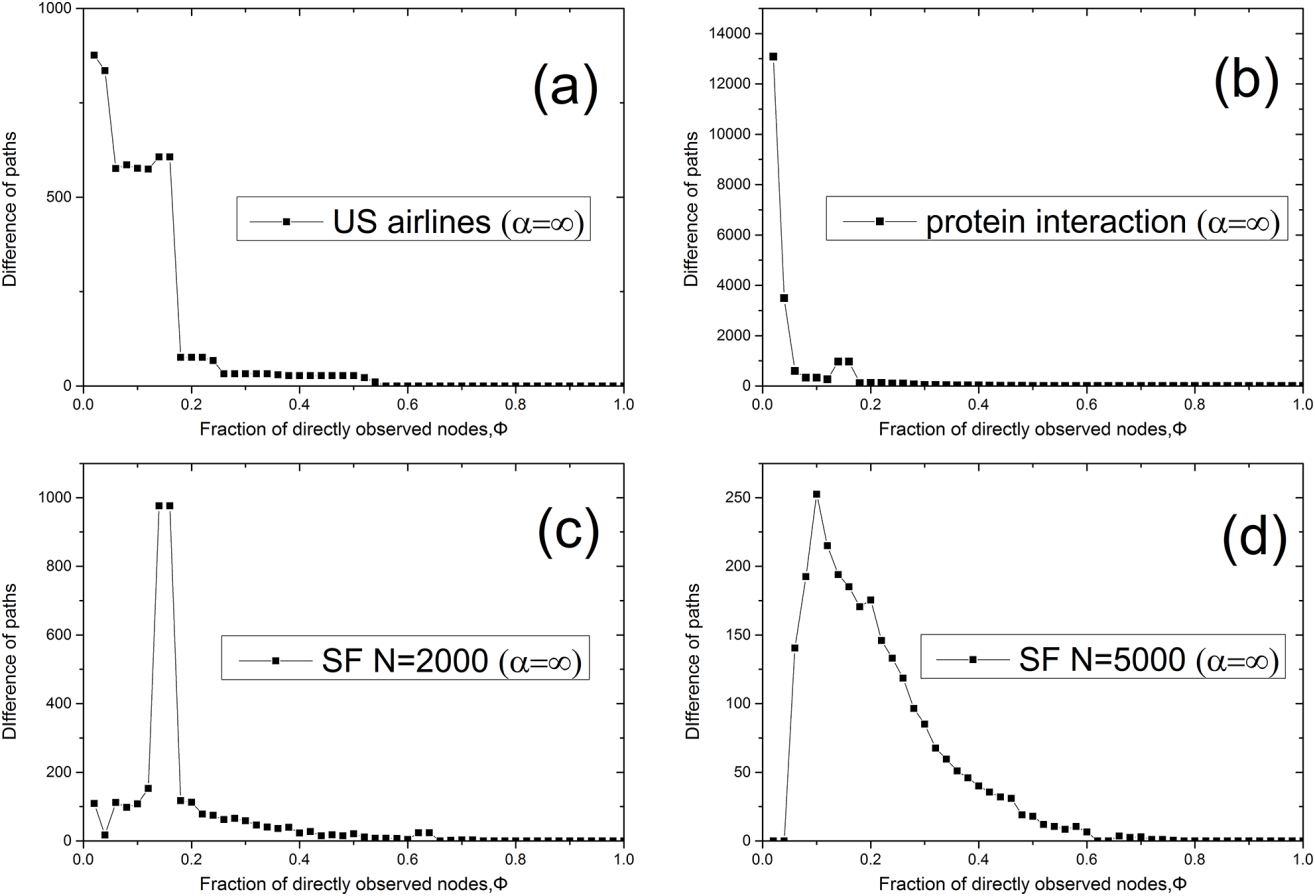
Difference of the number of paths via LOC (betweenness-degree). (*α* = ∞). (a) US airlines. (b) Protein interaction of Yeast. (c) Scale-free network with *γ* = 2.5, *N* = 2000. (d) Scale-free network with *γ* = 2.5, *N* = 5000.

Furthermore, LOC clusters generated by betweenness method have a significant role in network communication or traffic process. We calculate in Fig 6 the number of shortest paths passing through LOC in the network, suggesting that the betweenness method can generate a LOC containing more network load in model network and real networks. This feature allows LOC generated by betweenness method to observe more dynamical network traffic.

**Fig 7:**
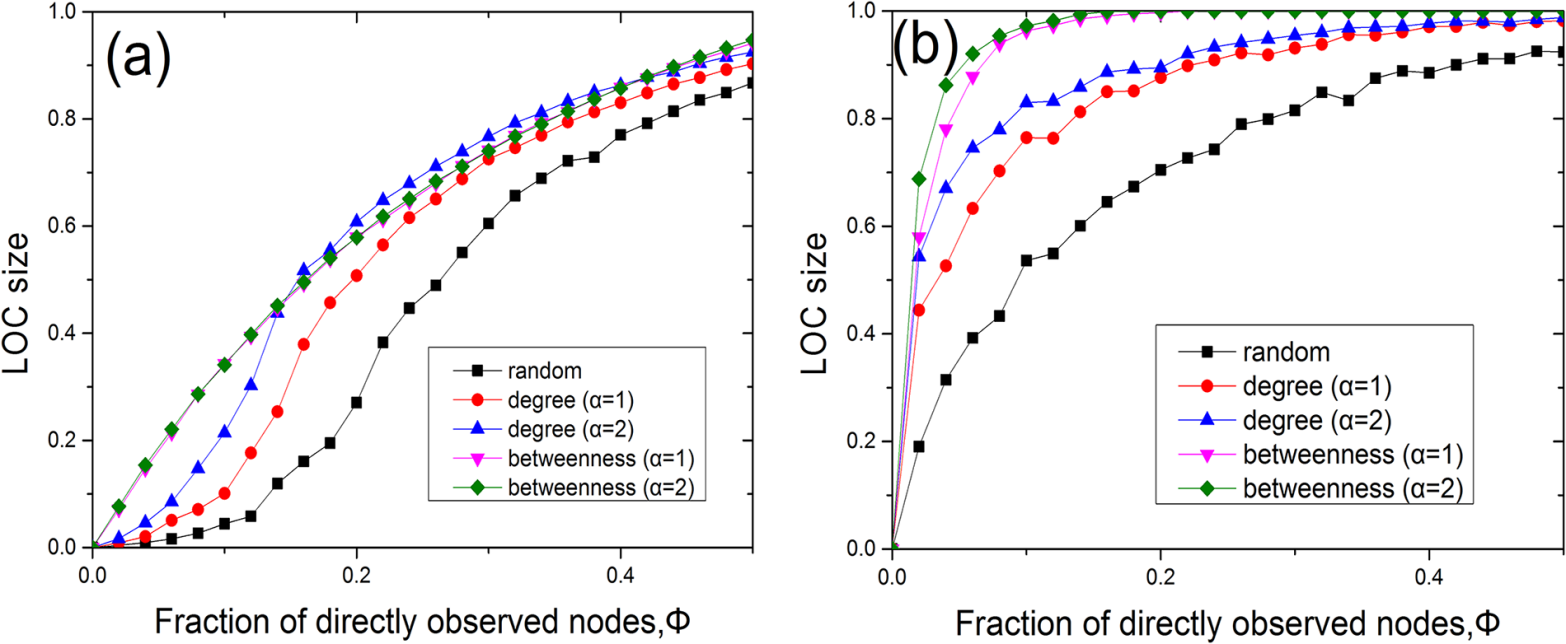
The LOC sizes in the power grid of the western United States and the network of US airlines. (a) The LOC size of the power grid of the western United States for random, degree-based and betweenness-based sensor placement. (b) The LOC size of the network of US airlines for random, degree-based and betweenness-based sensor placement.

To further study the observability transitions associated with betweenness-based placement, we analyze two real networks, namely, the power grid of the western United States [17] (Fig 7(a)) and the network of US airlines [18] (Fig 7(b)). The power grid of the western United States contains 4941 nodes and 6594 edges, with an average node degree of 〈*k*〉 = 2.669. The network of US airlines contains 332 nodes and 2126 edges, with an average node degree of 〈*k*〉 = 12.807. For the power grid network, the node degree distribution generally follows a Poissonian distribution, similar to that of an ER network, and the observability transition behaves smoothly, such that the LOC sizes attained using the degree-based method and the betweenness-based method are similar. By contrast, the airline network has a power-law degree distribution; as shown in Fig 7(b), the observability transition appears abrupt, as in the case of a scale-free network, and the LOC size for betweenness-based placement is larger than those for the other two methods. These findings suggest that the high-betweenness nodes are more essential for network observability in this case.

**Fig 8:**
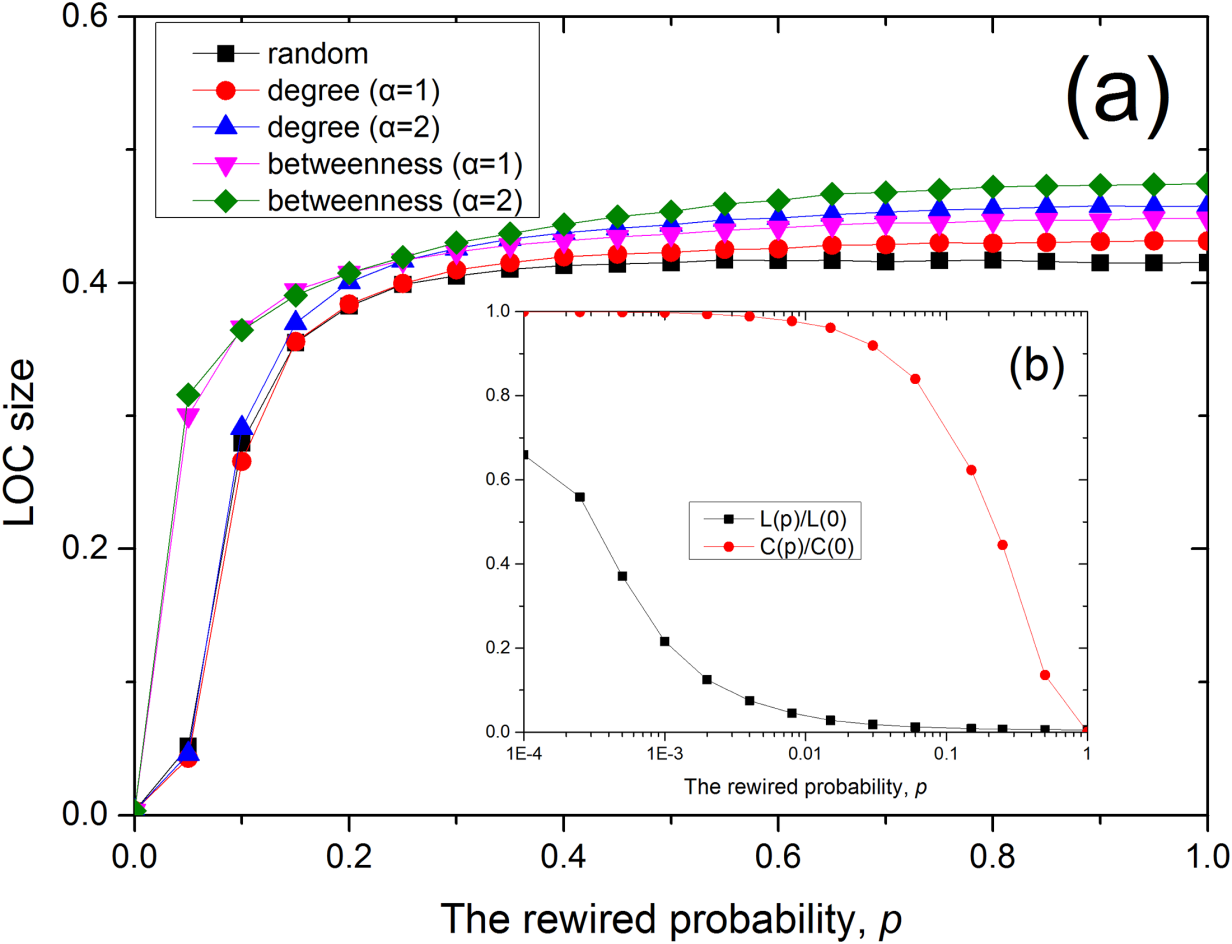
The LOC in WS network of *N* = 10^4^ and 〈*k*〉 = 4 for random, degree-based and betweenness-based sensor placement. (a)LOC size for Φ = 0.1. (b) *L(p)* is the characteristic path length and *C(p)* is the clustering coefficient under the given rewired probability *p*.

In addition, we also study the network observability on WS model [17] as Fig 8. In Fig 8, we find that small-world network under betweenness-based placement is helpful to the process of phase transition. This is because the small-world network with small clustering and small characteristic path length will increase the effect of high-betweenness nodes on the observability.

**Fig 9:**
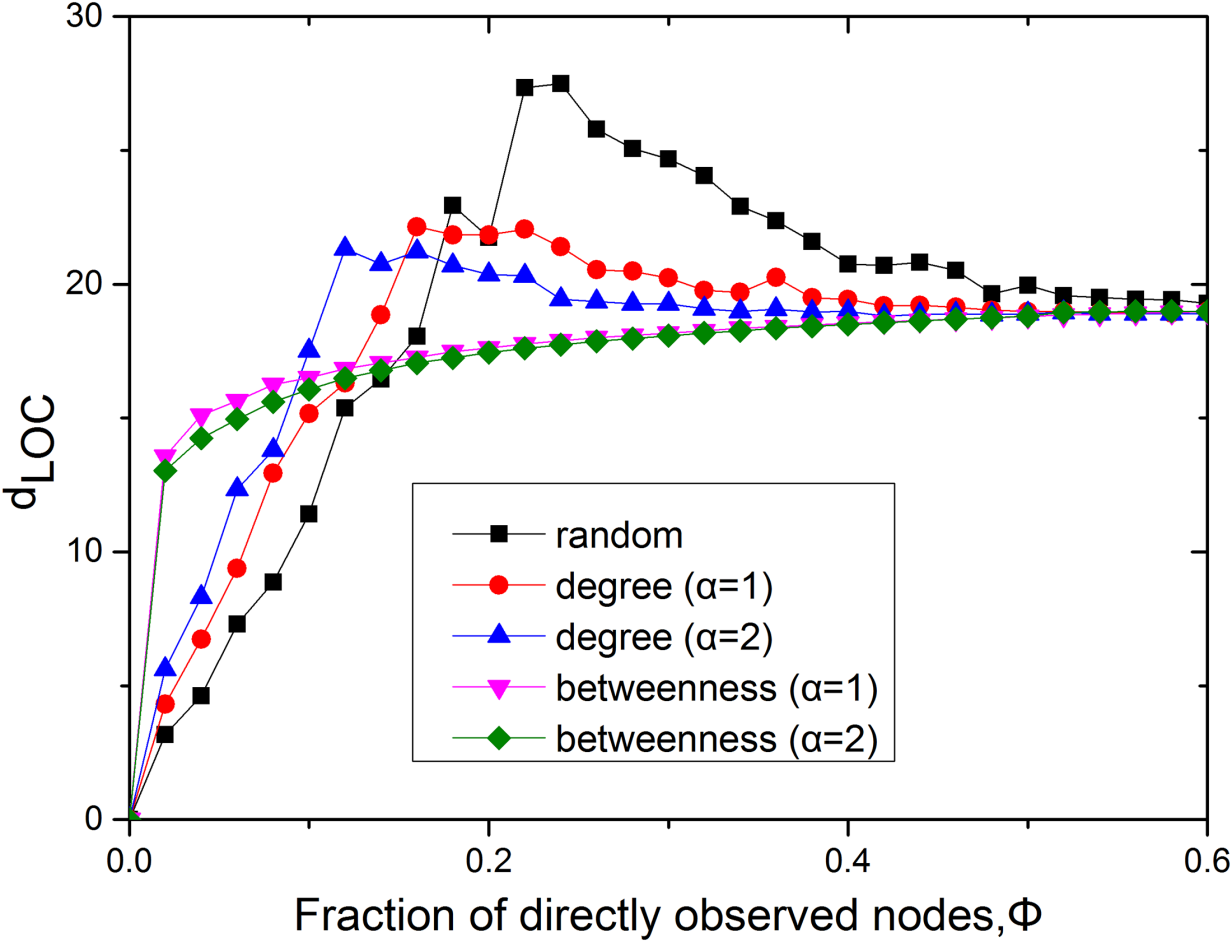
The LOC diameter in the power grid of the western United States. The black line shows the diameter of the LOC for random sensor placement, the red and blue lines show the diameter of the LOC for degree-based sensor placement, and the pink and green lines show the diameter of the LOC for betweenness-based sensor placement.

From Figs 2 and 7, it is evident that the nodes with high betweenness play an important role in the network observability. In particular, the size of the LOC is usually larger when sensors are placed in the network following the betweenness-based placement strategy. Nevertheless, regarding the relationship between betweenness and network observability, the internal structural properties of these LOCs remain unclear. For example, the diameter of the LOC quantifies the efficiency of communication inside the LOC. If the nodes in the LOC are far from each other, this may induce problems such as time delay, increased cost and even false estimation of the system state. Therefore, we should consider not only the size of the network’s observable component but also the diameter of the LOC. Fig 9 shows that the diameter of the LOC changes as a function of the fraction of nodes instrumented with sensors for all three placement strategies—random, degree-based and betweenness-based—in the power grid of the western United States. Initially, as sensors are added to the network, the diameter of the LOC should increase, corresponding to the expansion of the entire component. Past the threshold of the observability transition, the LOC has already extended to the full scale of the network and can be enlarged only by adding nodes that form alternative paths between existing nodes. In this stage, the diameter of the LOC will saturate or may even decrease with the addition of alternative routes between nodes. When a small fraction of the nodes are instrumented with sensors, the diameter of the LOC in the case of betweenness-based sensor placement is found to be larger than those for the other two methods. However, as the fraction of nodes with sensors increases, the diameter of the LOC for betweenness-based sensor placement becomes saturated and smaller than those for the other placements. This finding suggests that sensor placement based on betweenness can generate larger LOCs with smaller diameters.

## Conclusions

In this paper, to study how the observable clusters in network evolve when sensors are removed or damaged with decreasing Φ, we have examined the network observability transition for various topological methods of sensor placement. It has been demonstrated that in the case of betweenness-based sensor placement, the LOC is typically larger than the random or degree-based placement strategy in both models and real networks with a power-law degree distribution. And the betweenness-based method can generate a LOC containing more network load in model networks and real networks. Furthermore, when sensors are placed at a large fraction of the nodes, the LOC diameter in the case of betweenness-based sensor placement is significantly smaller than those for the other two methods in a real power grid. Considering that computational optimization methods for sensor placement typically require long computation times for large, complex networks, methods that incorporate topological information may provide a better understanding of the relationship between network observability and topology, which may assist in the design of improved optimization solutions.
